# Long-term surgical results of transposition of the great arteries with left ventricular outflow tract obstruction

**DOI:** 10.1186/s13019-022-01869-9

**Published:** 2022-05-11

**Authors:** Akihisa Furuta, Masaaki Yamagishi, Goki Matsumura, Takeshi Shinkawa, Hiroshi Niinami

**Affiliations:** Department of Cardiovascular Surgery, The Heart Institute of Japan, Tokyo Women’s Medical University, 8-1, Kawadacho, Shinjuku-ku, Tokyo, 162-8666 Japan

**Keywords:** Transposition of the great arteries, Left ventricular outflow tract obstruction, Double-outlet right ventricle

## Abstract

**Objective:**

The objective of this study was to evaluate the long-term surgical results of transposition of the great arteries with left ventricular outflow tract obstruction.

**Methods:**

We conducted a retrospective study of patients with transposition of the great arteries or double outlet right ventricle with left ventricular outflow tract obstruction undergoing biventricular repair between 1980 and 2017.

**Results:**

One hundred and eleven patients were enrolled and classified into five groups: atrial switch (n = 20), arterial switch (n = 12), Nikaidoh (n = 7), Rastelli (n = 48), and REV operation groups (n = 24). Early mortality was highest in Nikaidoh group (29%). Median follow-up was 18.2 years. Long-term survival was by far lowest in Nikaidoh group and comparable among the other 4 groups. Freedom from reoperation at 20 years was lowest in Rastelli group (32.1%) due to right ventricular outflow tract-related reoperations. While having no recurrence of left ventricular outflow tract obstruction, the arterial switch operation group had a high proportion of substantial neo-aortic regurgitation (29%).

**Conclusions:**

The long-term survival was satisfactory regardless of the surgical technique except Nikaidoh group. The surgical option for transposition of the great arteries with left ventricular outflow tract obstruction should be selected based on the features of the respective procedures.

## Introduction

The surgical treatment of transposition of the great arteries (TGA) with significant left ventricular outflow tract obstruction (LVOTO) continues to evolve. The Rastelli and atrial switch operations were first introduced as treatments for TGA with LVOTO in 1969 [1]. The réparation à l’etage ventriculaire (REV) operation to reconstruct the right ventricular outflow tract (RVOT) without using a prosthetic conduit was introduced by Lecompte in 1982 as an alternative for Rastelli candidates [2]. The aortic root translocation (Nikaidoh operation), first introduced in 1984 [3], has been recently proven to have the advantage of physiologic hemodynamics in the left ventricular outflow tract (LVOT) [4]. Although the presence of LVOTO has been considered to be a contraindication for the arterial switch operation (ASO) in the past, the surgical strategy has shifted in recent years toward aiming for an anatomically normal-shaped LVOT [5]. Additionally, the ASO with baffling of the ventricular septal defect (VSD) to the neo-aorta has become the treatment of double outlet right ventricle (DORV) with the anterior aorta, a left posterior overriding pulmonary artery (PA), and LVOTO. To achieve better long-term results, an understanding of the long-term features of these definitive operations for TGA and LVOTO is indispensable as guidelines for selecting the appropriate surgical option have not yet been established.

This study aims to compare the long-term results of the five main procedures: the atrial switch, arterial switch, Nikaidoh, Rastelli, and REV operations, and presented the long-term features of each procedure.

## Patients and methods

### Patient population and study design

This study is a retrospective, cohort study with consecutive patients undergoing biventricular repair for TGA or TGA-type DORV with LVOTO at the Tokyo Women’s Medical University Hospital from 1980 to 2017. During this period, a total of 748 patients were diagnosed with TGA or a TGA type of DORV and underwent surgery. Of these patients, 111 patients with TGA/LVOTO were enrolled herein. This study was approved and monitored by the Tokyo Women’s Medical University’s research ethics committee (institutional review board number: 4658) and the need for patient consent was waived due to the study’s retrospective nature. This study was performed in conformity with the Declaration of Helsinki. Medical records were reviewed, and the following data were retrieved and analyzed: basic demographic data, anatomical information, intraoperative data, and postoperative outcomes including catheter intervention, reoperation, and echocardiogram data.

In this study, we reviewed the transition of the surgical strategies for TGA with LVOTO, identified the advantages and disadvantages of the main five procedures by evaluating the long-term surgical results of TGA with LVOTO, and presented a perspective view of the strategies for TGA with LVOTO.

### Surgical procedure selection

**Fig. 1:**
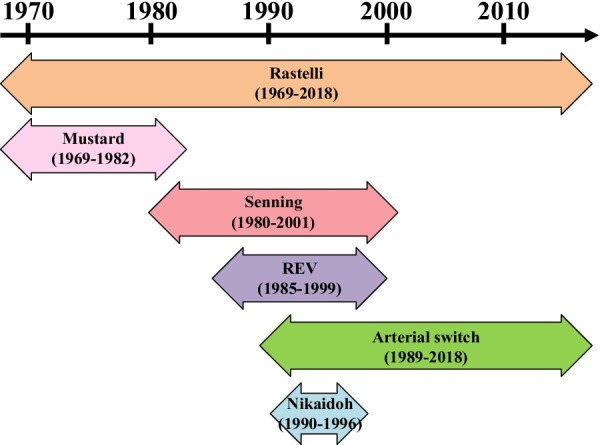
In the 1980s, the Senning and REV operations were introduced for the treatment of transposition of the great arteries with left ventricular outflow tract obstruction, in addition to the Rastelli and Mustard operations. the arterial switch and Nikaidoh operations were initiated in 1989 and 1990, however, the Nikaidoh procedure was abandoned in 1996 owing to the early mortality. The Senning and Mustard operations were discontinued in 1982 and 2001 owing to serious late complications. Subsequent attending surgeons opted to terminate the REV operation in 1999. The arterial and Rastelli operations had remained surgical options for this situation by 2017. REV, Réparation à l'Etage Ventriculaire

The type of definitive operation was selected for each patient based on the anatomic and hemodynamic characteristics during the study period (Fig. 1). The ASO and atrial switch operation were selected for the patients who had a relatively wide pulmonary annulus with no or small VSD. The atrial switch operation tended to be selected over the ASO for patients with unfavorable coronary artery patterns or with a high-pressure gradient on the LVOT (≥ 70 mmHg) until 2001 when the atrial switch operation was discontinued due to critical long-term complications. All corrections for the TGA-type DORV had been accomplished by the ASO. The Nikaidoh operation has been optimal for moderate LVOTO with a favorable coronary pattern since 1990. The Rastelli and REV operations have been adopted for severe LVOTO or pulmonary atresia and with preferable right ventricle volume and morphology. The choice between the Rastelli and REV operations depended on each surgeon’s assessment of the anatomic conditions: the Rastelli operation being preferred in patients with pulmonary artery distortion, multiple pulmonary artery stenosis, hypoplastic pulmonary artery, or elevated pulmonary vascular resistance.

### Statistical analysis and definition

All statistical analyses were performed with JMP Pro version 14 software (SAS Institute Inc., Cary, NC, USA). The normal or non-normal distribution of continuous variables was confirmed using the Shapiro–Wilk test. Data of continuous variables were presented as mean ± standard deviation for normal distribution and as median (25th–75th percentile interval) for non-normal distribution. Categorical variables were presented as a proportion (frequency). Early mortality was defined as death before hospital discharge or within 30 days of the definitive operation. The survival time was estimated from the date of the definitive operation to the date of all-cause death or the last contact, and the freedom from reoperation time was estimated from the date of definitive operation to the date of reoperation. The endpoint of follow-up was defined as the date of death or the last contact. Actuarial survival rates were analyzed by the Kaplan–Meier curve. Valve diameter was expressed as Z-scores [6]. LVOTO was classified into three types: subvalvular obstruction, valvular obstruction, and pulmonary atresia.

## Results

### Patient characteristics

A total of 111 patients were classified into five groups based on the type of definitive operations: the atrial switch operation (n = 20), ASO (n = 12), Nikaidoh (n = 7), Rastelli (n = 48), and REV operations (n = 24). The ASO group had the youngest mean operative age of 0.3 years and the Rastelli group had the oldest one of 5.3 years. Forty-six balloon atrial septostomy and 61 palliative operations were performed before the definitive operation. The category of LVOTO was valvular in 34 patients (31%), subvalvular in 30 (27%), atresia in 15 (14%), and subvalvular/valvular in 32 (29%). The most common type of LVOTO was small pulmonary diameter with valvular stenosis and left deviation of the infundibular septum with subvalvular stenosis. Preoperative median Z-scores of pulmonary diameter in valvular LVOTO for atrial switch operation, ASO, Nikaidoh operation, Rastelli operation, and REV operation groups were − 0.20 (range, − 0.4–0.6), 0.73 (range, − 0.5–1.7), − 0.64 (range, − 0.3–− 1.3), − 3.84 (range, − 14.0–− 1.2), and − 1.99 (range, − 6.4 to − 0.4), respectively. Preoperative left ventricular shortening fraction was not notably different between the groups. Preoperative patient data were summarized in Table 1.

**Table 1:** Patient characteristics

	Atrial switch n = 20	ASO n = 12	Nikaidoh n = 7	Rastelli n = 48	REV n = 24
Male	14 (70%)	10 (71%)	5 (71%)	25/48 (52%)	13 (54%)
Age (years)	1.2 (0.8–3.9)	0.3 (0.1–4.1)	3.4 (2.3–3.9)	5.3 (4.3–8.0)	3.8 (2.3–6.2)
Body weight (kg)	7.5 (5.7–12.6)	4.3 (3.5–12.8)	11.7 (10.3–12.3)	16.7 (13.4–21.4)	14.5 (10.1–16.1)
Type of LVOT obstruction Valvular Subvalvular Valvular and subvalvular Atresia	5 (25%) 13 (65%) 2 (10) None	1 (8%) 9 (75%) 2 (17%) None	5 (71%) 2 (29%) None None	16 (33%) 3 (6%) 17 (35%) 12 (25%)	7 (29%) 3 (13%) 11 (46%) 3 (13%)
Type of ventricular septal defect Intact septum Infundibular Perimembranous Muscular Multi-level	9 (50%) 1 (6%) 10 (50%) None None	1 (8%) 6 (50%) 3 (21%) 1 (8%) 1 (8%)	None 1 (14%) 6 (86%) None None	None 4 (8%) 38 (79%) None 6 (13%)	None 6 (25%) 14 (58%) None 4 (17%)
Bicuspid pulmonary valve	6 (30%)	2 (17%)	3 (43%)	8 (17%)	4 (17%)
Z-score of pulmonary diameter in valvular LVOTO	− 0.20 (− 0.21–0.19)	0.73 (0.63–0.87)	− 0.64 (− 0.45–− 0.91)	− 3.84 (− 4.82–− 3.43)	− 1.99 (− 2.74–− 0.35)
Pulmonary/aorta ratio in valvular LVOTO	0.83 ± 0.07	1.06 ± 0.05	0.65 ± 0.09	0.54 ± 0.13	0.71 ± 0.24
Fractional shortening	0.34 ± 0.09	0.38 ± 0.08	0.42 ± 0.04	0.37 ± 0.07	0.37 ± 0.06
Pressure gradient in LVOT (mmHg)	45 (30–56)	40 (27–63)	57.5 (35.8–66.5)	65 (58–79)	65 (47–73)

ASO, arterial switch operation; LVOT (O), left ventricular outflow tract (obstruction); REV, Réparation à l'Etage Ventriculaire

### Surgical technique

In atrial switch group, Senning (n = 18) or Mustard (n = 2) technique was used. In ASO group, the neo-PA was reconstructed by the original method (n = 2) or the Lecompte maneuver (n = 10), and the VSD was enlarged for intraventricular baffle in 5 TGA-type DORV patients. In Nikaidoh group, the RVOT was reconstructed using an autologous pericardial transannular patch. In Rastelli group, the RVOT was reconstructed with a xenograft roll conduit bearing handmade xenograft pericardial valves (1980–1996; n = 34), an autologous pericardial roll conduit bearing handmade autologous pericardial valves (1992–2012; n = 11), or an expanded polytetrafluoroethylene (e-PTFE) conduit with bulging sinuses and fan-shaped valves (2012–2017; n = 3) [7]. The conal flap method [8] was used in 20 patients with structural abnormalities of the tricuspid valve and the VSD was enlarged in 34 patients in this group. In REV group, the RVOT was reconstructed with a xenograft pericardial patch bearing a handmade xenograft pericardial valve (1980–1986; n = 6) or an autologous pericardial patch bearing a handmade autologous pericardial valve (1987–1999; n = 18). The mean perfusion and aortic cross-clamp times were longer in ASO (248 and 120 min) and Nikaidoh groups (242 and 125 min) and shortest in atrial switch group (138 and 79 min). In all groups, no patients were converted to other types of procedures. Operative results were summarized in Table 2.

**Table 2:** Operative results

	Atrial switch n = 20	ASO n = 12	Nikaidoh n = 7	Rastelli n = 48	REV n = 24
LVOTO relief Valvotomy Subvalvular muscle resection Both	4 (20%) 13 (65%) 2 (10%)	None 11 (92%) None	None None None	None 1 (2%) None	None None None
Concomitant procedure VSD closure Mitral valve repair Tricuspid valve repair COA repair Distal pulmonary artery angioplasty	11 (55%) 2 (10%) 1 (5%) None None	5 (42%) 2 (17%) None 2 (17%) None	None 1 (14%) None None 2 (29%)	None 1 (2%) 2 (4%) None None	None None None None None
Perfusion time (min)	138 ± 44	248 ± 32	242 ± 30	185 ± 46	176 ± 30
Aortic cross-clamp time (min)	79 ± 37	120 ± 34	125 ± 316	93 ± 36	94 ± 21

ASO, arterial switch operation; COA, coarctation of aorta; LVOT (O), left ventricular outflow tract (obstruction); REV, Réparation à l'Etage Ventriculaire; VSD, ventricular septal defect

### Overall survival

During the median follow-up of 18.2 (7.3–25.0) years, 74 patients were alive, 28 died, and 9 were lost to follow-up. There were 8 early mortalities (1 in atrial switch operation group [5%], 1 in ASO group [8%], 2 in Nikaidoh group [29%], and 4 in Rastelli group [8%]), which were all recorded in the 1980s or 1990s. Extracorporeal membrane oxygenation was introduced in four of these patients. There was no early mortality in REV group. The most frequent cause of these early mortalities was low output syndrome. The causes of death are shown in Table 3.

**Table 3:** The causes of death

	Atrial switch n = 20	ASO n = 12	Nikaidoh n = 7	Rastelli n = 48	REV n = 24
Early mortality Low output syndrome Bleeding	1 (5%) None None	1 (8%) 1 (8%) None	2 (29%) 2 (29%) None	4 (8%) 3 (6%) 1 (2%)	None None None
Late mortality, n (%) Sudden/unknown death Arrhythmia Heart failure Bleeding Cerebrovascular event Infection/Sepsis	3 (16%) 3 (16%) None None None None None	2 (18%) None None None None 1 (9%) 1 (9%)	2 (40%) 1 (20%) None None None None 1 (20%)	9 (20%) 4 (9%) 1 (2%) 1 (2%) 1 (2%) 1 (2%) 1 (2%)	4 (17%) 2 (8%) None 1 (4%) None None 1 (4%)

ASO, arterial switch operation; REV, Réparation à l'Etage Ventriculaire

**Fig. 2:**
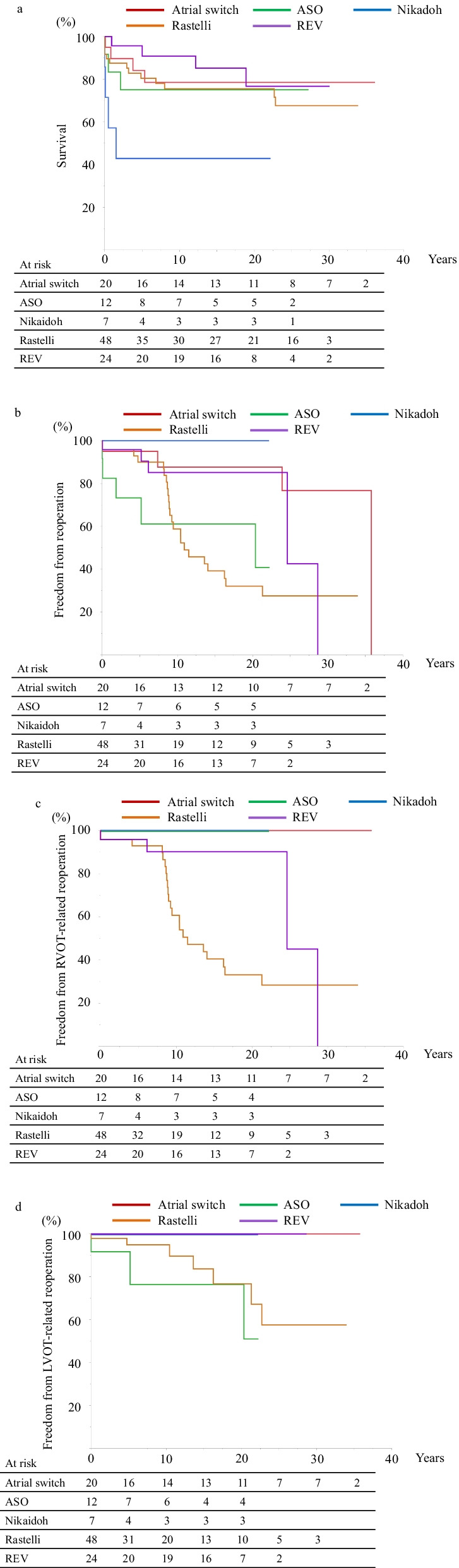
Kaplan–Meier survival curve. Kaplan–Meier survival curve is shown in the atrial switch operation (red), ASO (green), Nikaidoh (blue), Rastelli (orange), and REV (purple) groups. ASO, arterial switch operation; REV, Réparation à l'Etage Ventriculaire. **a** Survival. Survival at 20 years after definitive operation was 78.5 ± 9.6% in atrial switch operation group, 75.0 ± 12.5% in ASO group, 42.9 ± 18.7% in Nikaidoh group, 75.5 ± 6.5% (67.6 ± 7.9% at 30 years) in Rastelli group, and 76.7 ± 10.8% in REV group. **b** Freedom from reoperation. Freedom from reoperation at 20 years after definitive operation was 87.7 ± 8.3% (76.7 ± 12.6% at 30 years) in atrial switch operation group, 61.1 ± 15.7% in ASO group, 100% in Nikaidoh group, 32.1 ± 8.4% in Rastelli group (24.5 ± 8.4% at 30 years), and 85.2 ± 8.0% (42.6 ± 30.4% at 25 years) in REV group. **c** Freedom from RVOT-related reoperation. Freedom from RVOT-related reoperation at 20 years after definitive operation was 33.1 ± 8.9% in Rastelli group (28.4 ± 8.6% at 30 years), 90.2 ± 6.7% in REV group (42.6 ± 30.4% at 25 years), and 100% in the other groups. **d**. Freedom from LVOT-reoperation. Freedom from LVOT-related reoperation at 20 years after surgery was 76.4 ± 15.5% in ASO group, 76.6 ± 10.1% in Rastelli group (57.5 ± 13.9% at 30 years), and 100% in the other groups

Survival at 20 years after definitive operation was 78.5 ± 9.6% in atrial switch group, 75.0 ± 12.5% in ASO group, 42.9 ± 18.7% in Nikaidoh group, 75.5 ± 6.5% in Rastelli group, and 76.7 ± 10.8% in REV group (Fig. 2a). The deaths within three years after definitive operation were notable in ASO and Nikaidoh groups, however, there were no long-term mortalities in these groups. Mortalities more than10 years after definitive operation were recorded in the Rastelli and REV groups. The most frequent cause of late death was sudden/unknown reason, followed by infection/sepsis.

### Reintervention

Twenty-five patients (24%) required postoperative catheter intervention: 2 in atrial switch, 5 in ASO, 1 in Nikaidoh, 14 in Rastelli, and 3 in REV. The most common catheter intervention for each group was pacemaker implantation in atrial switch operation group (2/2: 100%), percutaneous RVOT dilatation in Rastelli group (8/14: 57%), and percutaneous distal PA angioplasty in ASO (5/5: 100%), Nikaidoh (1/1: 100%), and REV (2/3: 67%) groups. Catheter ablation was performed for supraventricular tachycardia in 3 patients in Rastelli group.

A total of 40 reoperations were conducted on 36 patients. Freedom from reoperation at 20 years after definitive operation was 87.7 ± 8.3% in atrial switch operation group, 61.1 ± 15.7% in ASO group, 100% in Nikaidoh group, 32.1 ± 8.4% in Rastelli group, and 85.2 ± 8.0% in REV group (Fig. 2b).

Thirty RVOT-related reoperations were conducted on 23 patients in Rastelli group (3 patients required twice) and 4 in REV group. In Rastelli group, conduit stenosis was repaired by conduit replacement (n = 20) or direct right ventricle-to-PA anastomosis with patch angioplasty (n = 2), and infectious endocarditis was treated by bioprosthetic pulmonary valve replacement (n = 1). In REV group, RVOT was reconstructed with an autologous pericardial patch bearing a handmade autologous pericardial valve (n = 4). Overall, freedom from RVOT-related reoperation at 20 years after definitive operation was 33.1 ± 8.9% in Rastelli group, 90.2 ± 6.7% in REV group, and 100% in other groups (Fig. 2c). A xenograft pericardial conduit/patch for RVOT reconstruction had a higher chance to require RVOT-related reoperation than an autologous pericardial conduit/patch in Rastelli and REV groups (62% vs. 18% in Rastelli group, and 50% vs. 6% in REV group).

Ten LVOT-related reoperations were conducted in 10 patients: 3 in ASO group and 7 in Rastelli group. Aortic valve replacement for neo-aortic regurgitation was required for 3 patients in ASO group. Seven LVOT-related reoperations in Rastelli group comprised 5 cases of re-intraventricular rerouting for intraventricular route stenosis and 2 of aortic valve replacement for aortic regurgitation. Of these 7 reoperations, 5 were performed concomitantly with RVOT-related reoperations. Conversely, there were no LVOT-related reoperations in REV group. Freedom from LVOT-related reoperation at 20 years after definitive operation was 76.4 ± 15.5% in ASO group, 76.6 ± 10.1% in Rastelli group, and 100% in other groups (Fig. 2d).

Other types of reoperations included 2 mitral valvuloplasties (1 in ASO and 1 in REV), 1 residual VSD closure with concomitant tricuspid annuloplasty (ASO), and 3 tricuspid valve replacement for systemic atrioventricular valve regurgitation (atrial switch operation).

### Echocardiographic data and clinical condition

Late echocardiography data were obtained from 73 of 83 survivors (88%). The median Z-score of valve diameter in the systemic ventricular outflow tract in atrial switch operation, ASO, Rastelli and REV groups was 2.0 (1.4–2.6), 1.7 (1.3–1.9), 0.2 (0.2–0.4), 2.5 (1.4–3.6), and 2.5 (1.4–3.6) respectively and the median flow velocity in the systemic ventricular outflow tract was 1.3 (1.1–1.4), 2.0 (1.6–2.1), 1.0 (0.9–1.1), 1.3 (0.9–2.0), and 1.3 (0.9–1.7) m/s respectively. A high incidence of substantial neo-aortic regurgitation was found in ASO group (2/7: 29%). A proportion of the substantial pulmonary regurgitation was comparable among Nikaidoh, Rastelli, and REV groups (67% vs. 58% vs. 63%). The median systemic ventricular shortening fraction in atrial switch operation, ASO, Nikaidoh operation, Rastelli operation, and REV operation groups was 0.20 (0.17–0.22), 0.37 (0.31–0.38), 0.31 (0.26–0.33), 0.28 (0.24–0.34), and 0.27 (0.26–0.29), respectively. No patients had Class III or IV on the New York Heart Association functional classification.

## Discussion

Since the initial atrial switch operation [9] and Rastelli operation [1] for TGA/LVOTO in 1969, several procedures have been developed, including the REV operation [2], aortic root translocation (Nikaidoh operation) [3], ASO, and their modifications, to treat this complex disease. For better long-term results, consideration of long-term features of respective operations is of great importance in selecting the definitive operation for TGA/LVOTO based on the morphology.

### Choices between ASO and Rastelli/REV/Nikaidoh

The surgical selection for TGA with LVOTO greatly changed after the introduction of the ASO, however, its use remains controversial. A relatively wide pulmonary annulus with no or small VSD made the ASO and atrial switch operation possible in our study. A reasonable threshold of the ASO (pulmonary valve Z-score of more than − 0.5) provided a favorable outcome with the mean late neo-aortic Z-score of 1.7 without LVOTO recurrence. However, our threshold of the indication for ASO might be extended to a pulmonary diameter Z-score of between − 1.5 and − 1.0 as a preoperative pulmonary valve Z-score of less than − 1.7 has been reported to be an independent predictor of recurrent LVOTO after the ASO for TGA/LVOTO [5]. Bibliographically, the Nikaidoh would be proposed for a Z-score of the pulmonary valve from − 1.5 to − 3.0 [4, 12], and the Rastelli and REV operations for a pulmonary diameter Z-score about less than − 3.0 [13].

### Comparison of Nikaidoh/Rastelli/REV

The Nikaidoh operation is a technically demanding operation with the risks of destabilizing the aortic valve or kinking/compressing the proximal coronary arteries. Due to frequent coronary troubles, the Nikaidoh operation was abandoned in 1996 at our institution. Nikaidoh group in our study experienced the highest early mortality and consequently the lowest long-term survival of 42.9% among the groups while presenting with a low rate of late mortality and reoperation. The Nikaidoh operation has been reported to provide low early mortality of 3–5% as well as superior long-term outcomes in some reports [4, 14]. Additionally, half truncal switch operation, which overcomes various drawbacks of the conventional operations and takes on all the advantages of the Nikaidoh operation, recently presented with excellent long-term results [13]. These types of operations, therefore, would become the mainstream for TGA with moderate LVOTO. As for other types of operations, Rastelli and REV groups presented with reasonable long-term survival of about 76% in our study, which is similar to or relatively higher than previous reports [15–17], however, there remains a concern about late death in Rastelli and REV group.

RVOT obstruction or pulmonary regurgitation is the most serious complication after the definitive operation requiring RVOT reconstruction. Similar to previous reports [15, 16, 18], a high incidence (48%) of RVOT-related reoperation was documented in Rastelli group in our study, which were all performed among patients where xenograft or autologous pericardial conduits were used, while there were no RVOT-related reoperations among patients where e-PTFE conduits used. Considering the excellent long-term results for RVOT reconstruction by e-PTFE conduits [7], this might be expected to contribute to improving the result after the Rastelli operation. On the other hand, our study showed REV group had a significantly lower proportion of RVOT- and LVOT-related reoperations than Rastelli group, which would be achieved based on the distinctive characteristic of avoiding the negative effects of both RVOT and LVOT obstruction [19]. Although the REV operation has regrettably been discontinued in 1999 at our institution by incoming attending surgeons despite the favorable outcomes, this operation could be one of the optimal procedures for patients with severe LVOTO unsuitable for ASO and aortic root translocation.

### Aortic valve function in ASO group

The presence of LVOTO in TGA patients was revealed to be an independent risk factor after the ASO [5, 20, 21]. Substantial neo-aortic regurgitation was actually found in 29% of survivors in our ASO group, which seemed higher than after ASO in simple TGA [21]. It is presumed that the pulmonary valve, which is protected by a low pressure before the ASO, reacts to the sudden onset of arterial pressure by a dilatory response. Additionally, the leaflets may be damaged in the course of LVOTO relief and transpulmonary VSD closure [21]. Although our study lacks data regarding a bicuspid pulmonary valve owing to mortality, it seems to be associated with late aortic root dilatation and aortic regurgitation [22, 23].

### Limitations

This study has certain limitations: this was a retrospective, single-center study and biases were present owing to a lack of homogeneity, which can make group comparisons inaccurate; and finally, there was a lack of cases with follow-up. As this was a long and wide retrospective study, our study also included bias regarding postoperative management, surgical techniques, and uncommon surgical strategies.

## Conclusions

The 40-year surgical results at our institution revealed the unique advantages and disadvantages of the respective procedures. There were no significant differences in survival between the groups except for Nikaido group. Although having a low incidence of LVOTO recurrence and the best systemic ventricular function, ASO group presented a high proportion of late substantial neo-aortic regurgitation. In terms of avoidance of reoperation, the REV operation could be one of the optimal procedures for patients with severe LVOTO unsuitable for ASO and aortic root translocation.

## Data Availability

The datasets used and/or analyzed during the current study are available from the corresponding author on reasonable request.

## Abbreviations

ASO: Arterial switch operation
DORV: Double outlet right ventricle
e-PTFE: Expanded polytetrafluoroethylene
LVOT: Left ventricular outflow tract
LVOTO: Left ventricular outflow tract obstruction
PA: Pulmonary artery
REV: Réparation à l’etage ventriculaire
RVOT: Right ventricular outflow tract
TGA: Transposition of the great arteries
VSD: Ventricular septal defect
